# Prostaglandin E_2_ induces DNA hypermethylation in gastric cancer *in vitro* and *in vivo*

**DOI:** 10.7150/thno.35766

**Published:** 2019-08-14

**Authors:** Chi Chun Wong, Wei Kang, Jiaying Xu, Yun Qian, Simson Tsz Yat Luk, Huarong Chen, Weilin Li, Liuyang Zhao, Xiaoming Zhang, Phlip WY Chiu, Enders KW Ng, Jun Yu

**Affiliations:** 1 Institute of Digestive Disease and Department of Medicine and Therapeutics, State Key Laboratory of Digestive Disease, Li Ka Shing Institute of Health Sciences, CUHK-Shenzhen Research Institute, The Chinese University of Hong Kong, Hong Kong.; 2 Department of Anatomical and Cellular Pathology, The Chinese University of Hong Kong, Hong Kong; 3 Department of Gastroenterology, Shenzhen University General Hospital, Shenzhen University Clinical Medical Academy, Shenzhen, China; 4 Department of Surgery, The Chinese University of Hong Kong, Hong Kong

**Keywords:** Gastric cancer, COX-2 transgenic mice, Prostaglandin E_2_, DNA methylation, DNMT3B

## Abstract

**Rationale**: Prostaglandin E_2_ (PGE_2_) is a pro-inflammatory eicosanoid up-regulated in gastric cancer (GC). However, its impact on epigenetic dysfunction in the process of gastric carcinogenesis is unknown. In this study, we investigate the role of PGE_2_ in DNA methylation in gastric epithelium *in vitro*, in mice, and humans.

**Methods**: PGE_2_-induced DNMT3B and DNA methylation was determined in gastric cell lines and COX-2 transgenic mice. Effect of COX-2 inhibition on DNA methylation was evaluated in a randomized controlled trial. Efficacy of combined COX-2/PGE_2_ and DNMT inhibition on GC growth was examined in cell lines and mice models.

**Results**: PCR array analysis of PGE_2_-treated GC cells revealed the up-regulation of DNMT3B, a *de novo* DNA methyltransferase. In GC cells, PGE_2_ induced DNMT3B expression and activity, leading to increased methylated cytosine (5mC) and promoter methylation of tumor suppressive genes (MGMT and CNR1). Consistently, Cox-2 (rate-limiting enzyme for PGE_2_ biosynthesis) transgenic expression in mice significantly induced Dnmt3b expression, increased 5mC content, and promoted Mgmt promoter methylation. We retrospectively analyzed the 5mC content of 42 patients with intestinal metaplasia (a precancerous lesion of GC) treated with a COX-2 specific inhibitor Rofecoxib or placebo for 2 years, revealing that the COX-2 inhibitor significantly down-regulated 5mC levels (N=42, P=0.009). Collectively, these data indicate that PGE_2_ is closely related to DNA hypermethylation *in vitro* and *in vivo*. Using genome-wide 450K methylation array, we identified chromosomal genes (POT1, ATM and HIST1H2AA) were preferentially methylated by PGE_2_. Biofunctional work revealed that POT1 functions as a tumor suppressor. Finally, we demonstrated that combinatorial inhibition of COX-2 and DNMT using Celecoxib and Decitabine synergistically inhibited GC growth *in vitro* and *in vivo*.

**Conclusion**: This study suggested that PGE_2_ promotes DNA methylation in GC, and that co-targeting of PGE_2_ and DNMT inhibits GC.

## Introduction

Gastric cancer (GC) is the fourth most common cancer and the second leading cause of cancer deaths worldwide 1, 2. GC is prevalent in East Asia, such as Japan and China 3. Cyclo-oxygenase-2 (COX-2), the rate-limiting enzyme for biosynthesis of prostaglandin E2 (PGE_2_), is frequently over-expressed in *Helicobacter pylori* infected-patients and in patients with gastric precancerous lesions or GC 4, 5. COX-2 promotes carcinogenesis by supporting the production of PGE_2_. PGE_2_ is a potent pro-inflammatory eicosanoid transducing its signals via E-prostanoid receptors 1-4 (EP 1-4), and PGE_2_ has a key role in tumorigenesis by inducing sustained inflammation, and promoting cell proliferation, angiogenesis, invasion and metastasis 4, 6, 7.

Aberrant promoter DNA methylation is a hallmark of GC. We previously identified tumor suppressor genes such as SCNN1B and CAB39L 8, 9 that are silenced by promoter DNA methylation in GC patients. However, the underlying cause of this epigenetic event in GC remains poorly understood. *H. pylori*-induced inflammation is indispensable for DNA methylation 10, indicating that pro-inflammatory factors such as COX-2/PGE_2_ axis may contribute to epigenetic dysregulation.

COX-2 is a well-established chemoprevention target for cancers 11. Targeting of COX-2 using non-steroidal anti-inflammatory drugs (NSAIDs) have shown promising efficacy in GC 12. NSAIDs suppress gastric cancer cell growth and effectively prevent GC in animal models 11, 13. Epidemiological data support the use of NSAIDs in GC prevention 14, 15. Nevertheless, NSAIDs use only in GC is limited by its efficacy. Combinatorial strategies may provide a dramatic improvement over single agents 16. Given the potential role of PGE_2_ in epigenetic dysregulation in cancers, we reasoned that combining COX-2 inhibitors and epigenetic drugs may be synergistic in inhibiting cancer initiation and progression.

Here, we established a role for COX-2/PGE_2_ in DNA methylation in GC via up-regulating DNMT3B, leading to aberrant DNA methylation. This observation is consistent in gastric cell lines and COX-2 transgenic mice. Corroborating the preclinical results, retrospective analysis of patients with intestinal metaplasia revealed that COX-2 inhibitor treatment reduced DNA methylation. Genome-wide methylation profiling further identified PGE_2_-induced driver methylation genes that contributes to carcinogenesis. Finally, we showed that the concomitant inhibition of COX-2 and DNMT was synergistic in the suppression of GC growth.

## Results

### PGE_2_ induced DNMT3B expression and activity in GC cell lines

**Figure 1:**
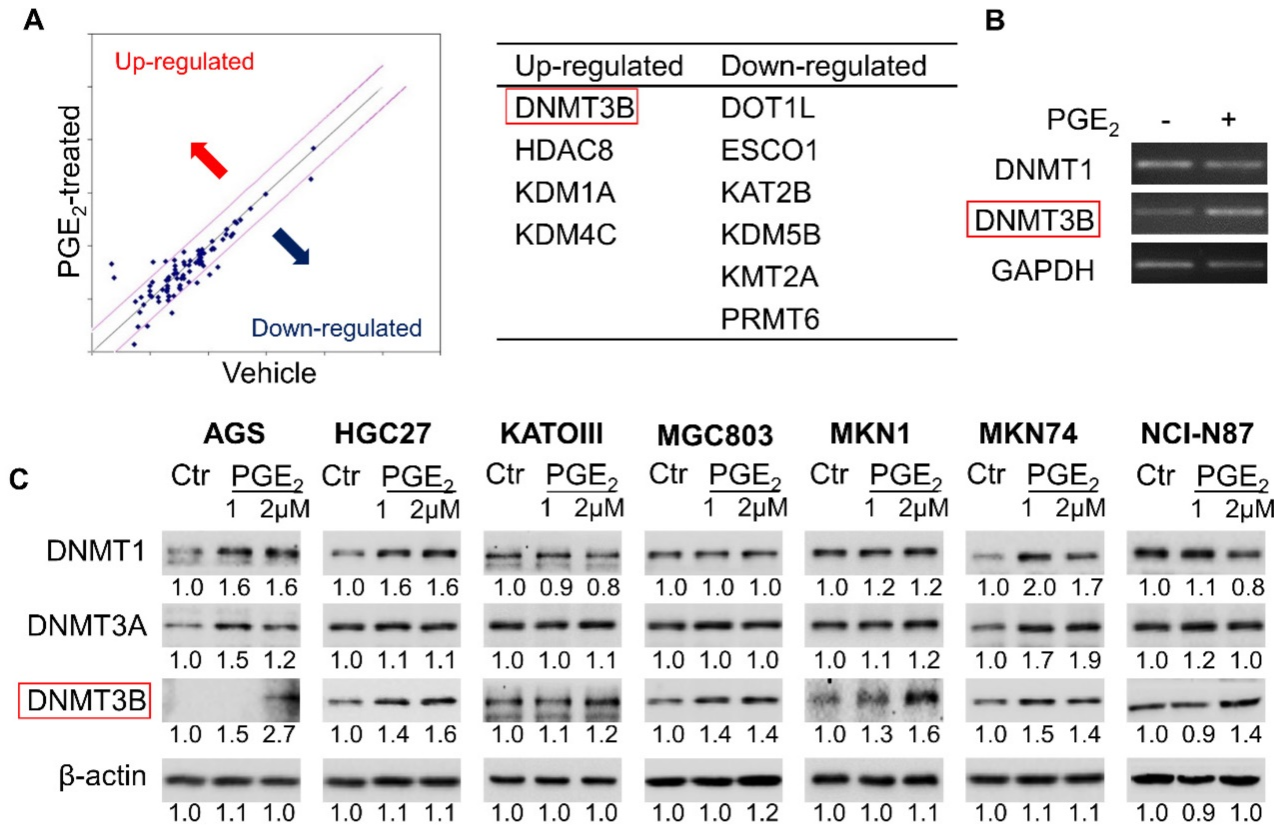
PGE_2_ induced expression of DNA methyltransferase 3B (DNMT3B) in gastric cancer. (a) Epigenetic Chromatin Modification Enzymes PCR array analysis of PGE_2_-treated KATOIII cells revealed alteration of various epigenetic regulators. (b) RT-PCR analysis of DNMT1 and DNMT3B mRNA expression after PGE_2_ treatment in KATOIII cells. (c) Western blot showed that PGE_2_ uniformly induced DNMT3B, but not DNMT1 or DNMT3A protein in GC cell lines. The relative expression was determined by densitometry analysis (Image Lab Software).

**Figure 2:**
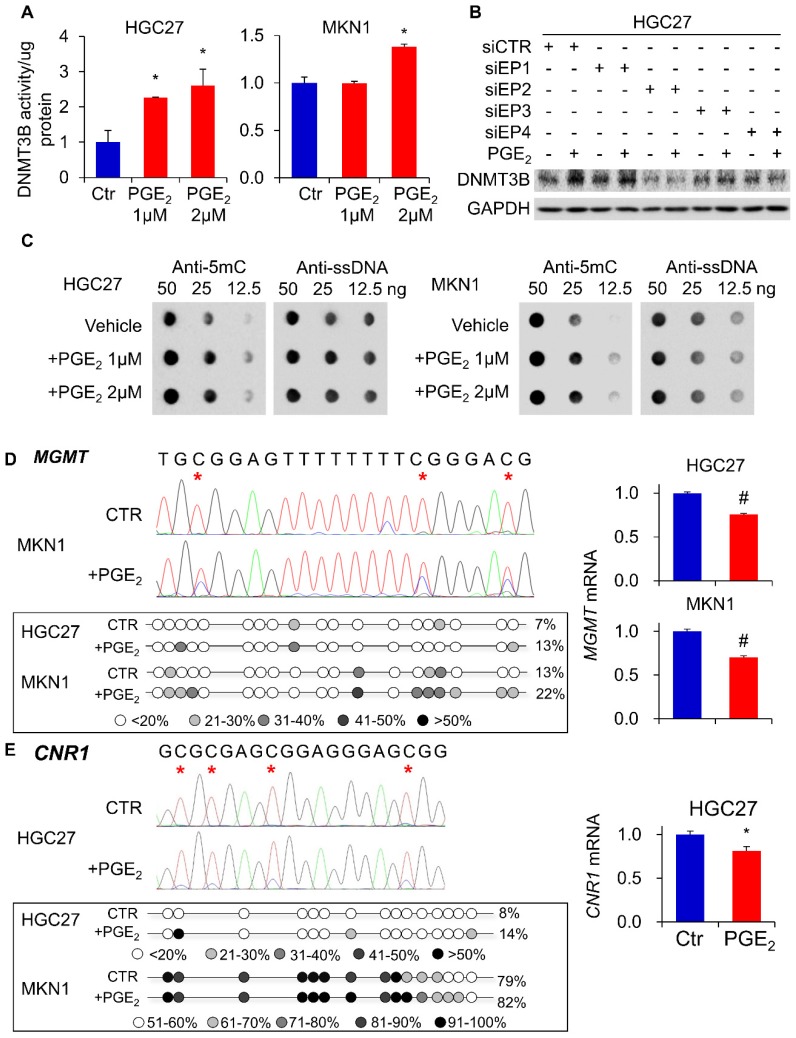
PGE_2_ induced DNMT3B activity, 5mC levels and promoter DNA methylation. (a) PGE_2_ increased nuclear DNMT3B activity in HGC27 and MKN1 cells, as determined by DNMT3B assay kit. (b) Knockdown of EP2 or EP4 in HGE27 cells abrogated the induction of DNMT3B expression by PGE_2_. (c) PGE_2_ promoted 5mC levels in HGC27 and MKN1 cells, as indicated by dot blot assay (d) PGE_2_ induced promoter methylation of O^6^-Methylguanine-DNA Methyltransferase (MGMT) in gastric cancer cells, leading to down-regulation of MGMT mRNA expression. (e) PGE_2_ induced promoter methylation of cannabinoid receptor type 1 (CNR1) in HGC27 and MKN1 cells as determined by BGS. CNR1 mRNA was also down-regulated by PGE_2_ in HGC27 cells. *MGMT* and *CNR1* promoter methylation were determined by Bisulfite sequencing.

To identify epigenetic enzymes altered by PGE_2_, we evaluate the profiled expression of a gastric cancer cell (KATOIII) treated with vehicle or 2µM PGE_2_ for 48h with Epigenetic Chromatin Modification Enzymes PCR array. We have identified differentially expressed genes involved in epigenetic regulation (**Figure 1A**) such as DNA methylation (DNMT3B), histone methylation (DOT1l and PRMT6), histone demethylation (KDM1A, KDM4C and KDM5B), histone acetylation (ESCO1 and KAT2B) and histone deacetylation (HDAC8). RT-PCR confirmed up-regulation of DNMT3B mRNA in KATOIII cells treated with PGE_2_ (**Figure 1B**). We investigated the effect of PGE_2_ on protein expression of DNMT1, DNMT3A, and DNMT3B in a panel of GC cell lines. As shown in **Figure 1C**, PGE_2_ consistently induced DNMT3B expression in all GC cell lines tested. PGE_2_ also induced DNMT1 and DNMT3A expression in AGS and MKN74 cells, but it had varied effects on the other cell lines, suggesting cell-line dependent effects. DNMT3B possesses '*de novo*' activity that establishes DNA methylation pattern, and it has been shown to be induced by PGE_2_ in colorectal cancer 16. Effect of PGE_2_ is dose-dependent, as the highest dose of PGE_2_ (2µM) more consistently induced expression of DNMT3B. We next evaluated nuclear DNMT3B activity in HGC27 and MKN1 cell lines (**Figure 2A**), which have demonstrated the strongest up-regulation of DNMT3B. In both GC cell lines, treatment with PGE_2_ for 48h dose-dependently increased the nuclear DNMT3B activity (*p*<0.05), suggesting that PGE_2_ could promote DNA methylation by inducing expression and activity of DNMT3B. PGE_2_ transduces its signal via EP receptors (EP1-4). To evaluate whether PGE_2_ induces expression of DNMT3B via EP receptors, we performed siRNA mediated knockdown of EP1-4 in HGC27 cells (**Supplementary Figure 1**) and evaluated PGE_2_-induced DNMT3B protein expression. Knockdown of EP2/4 reduced PGE_2_-induced DNMT3B expression, whereas knockdown of EP1/3 had no effect, suggesting that EP2 and EP4 mediate effect of PGE_2_ on DNMT3B expression (**Figure 2B**).

### PGE_2_ induced 5-methylcytosine (5mC) levels and enhanced promoter methylation of *O*-6-methylguanine-DNA methyltransferase (*MGMT*) and cannabinoid receptor type 1 (*CNR1*)

To evaluate whether DNMT3B up-regulation translates to an increased DNA methylation, we examined overall levels of 5mC in HGC27 and MKN1 cells genomic DNA. As shown in **Figure 2C**, PGE_2_ treatment induced 5mC levels in both cell lines, as determined by dot blot assay (total ssDNA as loading control), indicating that PGE_2_ increased overall DNA methylation. We next tested whether PGE_2_ induced promoter hypermethylation. *MGMT* and *CNR1* are two classical markers for the CpG island methylator phenotype (CIMP) and they are hypermethylated in cancers 16. We therefore examined their promoter methylation status in HGC27 and MKN1 cells using bisulfite genomic sequencing (BGS). As shown in **Figure 2D**, we evaluated 19 CpGs at the *MGMT* promoter, and their showed increased methylation after treatment with PGE_2_. Over the CpG sites examined, their methylation was significantly increased in HGC27 (7 to 13%, *P*<0.01) and MKN1 (13 to 22%, *P*<0.01) cells. In line with its promoter methylation, mRNA expression of MGMT was reduced by PGE_2_ treatment in HGC27 and MKN1 cells. A similar phenomenon was observed in the *CNR1* promoter (**Figure 2E**), which was hypermethylated in HGC27 (8 to 14%, *P*<0.01) and MKN1 (79% to 89%, *P*<0.01) cells after PGE_2_ treatment. Silencing of CNR1 mRNA was induced by PGE_2_ in HGC27 cells; whereas CNR1 was not detected in MKN1 cells owning to its high endogenous promoter methylation. These findings indicated that PGE_2_ increased overall DNA methylation and also induced promoter methylation of tumor suppressor genes, consistent with enhanced activity of DNMT3B.

### Transgenic COX-2 expression in mice increased DNMT3B expression, 5-methylcytosine levels and *MGMT* promoter methylation

**Figure 3:**
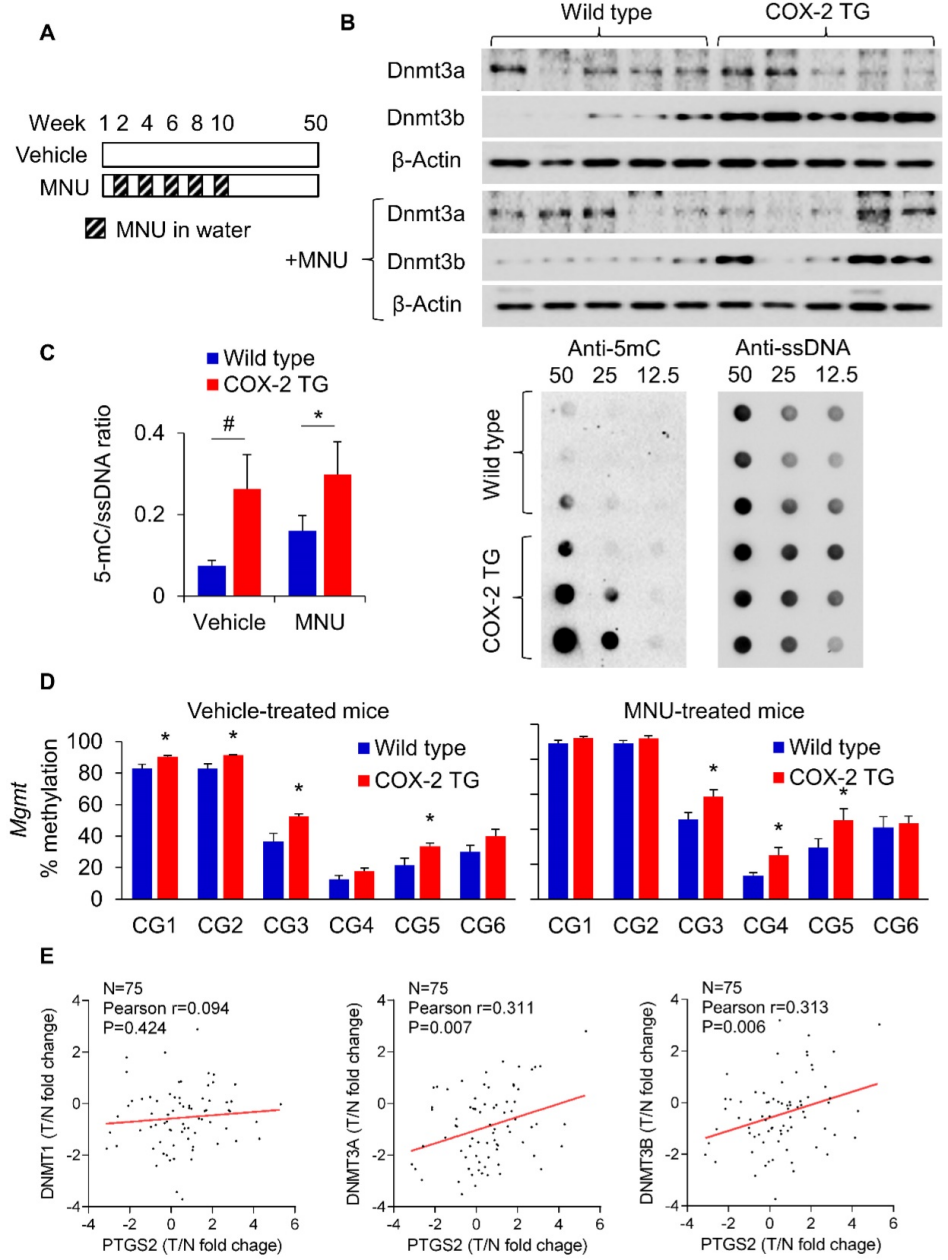
PGE_2_ induced DNMT3B expression and DNA methylation *in vivo*. (a) COX-2 transgenic mice were treated with water or MNU and gastric tissues were collected at 50 weeks. (b) Western blot showed that Dnmt3b, but not Dnmt3a, was to be overexpressed in mice with transgenic COX-2 overexpression. (c) Transgenic COX-2 mice revealed DNA hypermethylation, as evidenced by significantly increased 5mC levels (WT-vehicle, N=15; COX-2 TG-vehicle, N=11; WT-MNU, N=22; COX-2 TG-MNU, N=16). (d) Transgenic COX-2 mice showed increased *Mgmt* promoter as determined by BGS in both vehicle (WT, N=7; COX-2 TG, N=6) and MNU treatment groups (WT, N=13; COX-2 TG, N=11). (e) Correlation between levels of PTGS2 (COX-2), and DNMT1, DNMT3A and DNMT3B in human gastric cancer patients. PTGS2 mRNA is correlated with the expression levels of DNMT3A and DNMT3B (*right*), but not of DNMT1 (*left*).

To evaluate the *in vivo* relevance of PGE_2_ in DNMT3B expression and DNA methylation, we utilized a transgenic COX-2 mouse as previously reported (**Supplementary Figure 2 and Figure 3A**) 17. COX-2 transgenic mice showed a marked induction in the production of PGE_2_ 17. COX-2 transgenic and wild type mice were either treated with vehicle or MNU (for induction of GC). We first examined expression of Dnmt3a and Dnmt3b in these mice. Western blot showed that Dnmt3b protein was induced in COX-2 transgenic mice compared to wildtype (**Figure 3B**) in both vehicle- and MNU-treated groups. We next compared the overall 5mC levels in COX-2 transgenic mice and wildtype mice by dot blot assay (**Figure 3C**). COX-2 transgenic mice exhibited higher 5mC levels compared to wildtype mice independent of vehicle or MNU treatment. In both groups, COX-2 increased 5mC levels (*P*<0.05). Given that transgenic COX-2 expression in mice induced Dnmt3b, we tested its effect on promoter DNA methylation. BGS analysis of the *Mgmt* promoter demonstrated that COX-2 transgenic expression promoted methylation at multiple promoter CpG sites in vehicle and MNU-treated groups (*P*<0.05) (**Figure 3D**). MNU-treated COX-2 transgenic mice had increased methylation at CG3, CG4 and CG5 (P<0.05) compared to control COX-2 mice, suggesting that COX-2 overexpression might induce *Mgmt* promoter methylation during gastric carcinogenesis *in vivo*. Correspondingly, MGMT mRNA was suppressed in COX-2 transgenic mice (**Supplementary Figure 3**). These data indicate that transgenic COX-2 expression in mice is associated with increased DNMT3B expression, 5mC levels and *Mgmt* promoter hyper-methylation, leading to transcriptional silencing of MGMT. To determine the clinical relevance of PGE_2_ in induction of DNMTs in humans, we examined the correlation between the expression of DNMT1/DNMT3A/DNMT3B and COX-2 in a Hong Kong cohort of 75 GC cases (**Figure 3E**). We observed a positive correlation between DNMT3A (*P*=0.007) and DNMT3B (*P*=0.006) with COX-2, but not for DNMT1 (*P*=0.424). The positive correlations between DNMT3A/DNMT3B with PTGS2 was validated in an independent gastric cancer cohort (GSE27342, **Supplementary Figure 4**). Collectively, these findings indicate that the COX-2-PGE_2_ axis promotes DNA methylation *in vitro* and* in vivo*.

### A COX-2 specific inhibitor suppressed DNA methylation in humans

**Figure 4:**
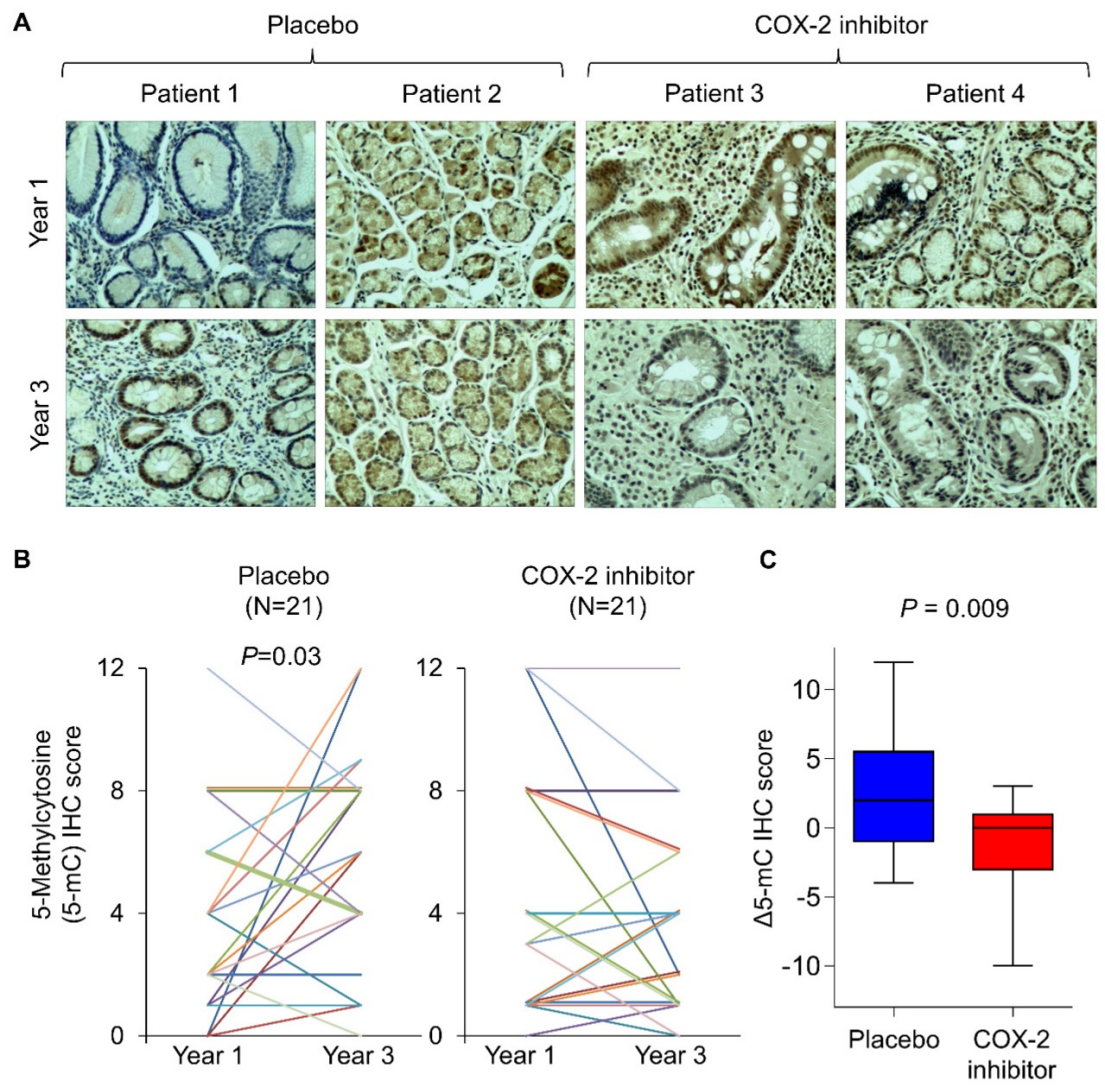
COX-2 specific inhibitor slowed the elevation of 5mc levels in patients with intestinal metaplasia. (a) Representative IHC staining of 5mC in gastric tissues from intestinal metaplasia patients treated with placebo or rofecoxib for 2 years. (b) IHC scoring by a pathologist at baseline and year 3 revealed that placebo group had significantly increased 5mC score, but no statistically significant difference was shown in rofecoxib group. (c) Alteration in the 5mC levels between year 1 and 3 (Δ5mC) was significant decreased in rofecoxib group as compared to placebo.

To corroborate the findings in humans, we retrospectively analyze tissue biopsies from a randomized, placebo-controlled trial involving long-term treatment with a COX-2 inhibitor in patients with gastric intestinal metaplasia, a precancerous condition 18. Forty-two age- and gender-matched patients from vehicle (N=21) and rofecoxib (N=21) group. To analyze DNA methylation in tissue biopsies, we performed immunohistochemistry with anti-5mC and determined its nuclear staining score at baseline and two years after the initiation of drug treatment (**Figure 4A**). Representative images suggest that 5mC levels in placebo group had increased 5mC levels after 2 years, which was statistically significant (**Figure 4B**,* P*<0.05). In contrast, rofecoxib group exhibited no elevation of 5mC levels (**Figure 4B**). Comparing the change in 5mC (Δ5mC) at year 2 to baseline, rofecoxib was found to significantly reduce Δ5mC compared to placebo (**Figure 4C**, *P*=0.009). Thus, COX-2/PGE_2_ blockade might slowed progressive hypermethylation in patients with intestinal metaplasia.

### Genome wide profiling of PGE_2_-induced DNA methylation revealed gene-specific methylation induced by PGE_2_

**Figure 5:**
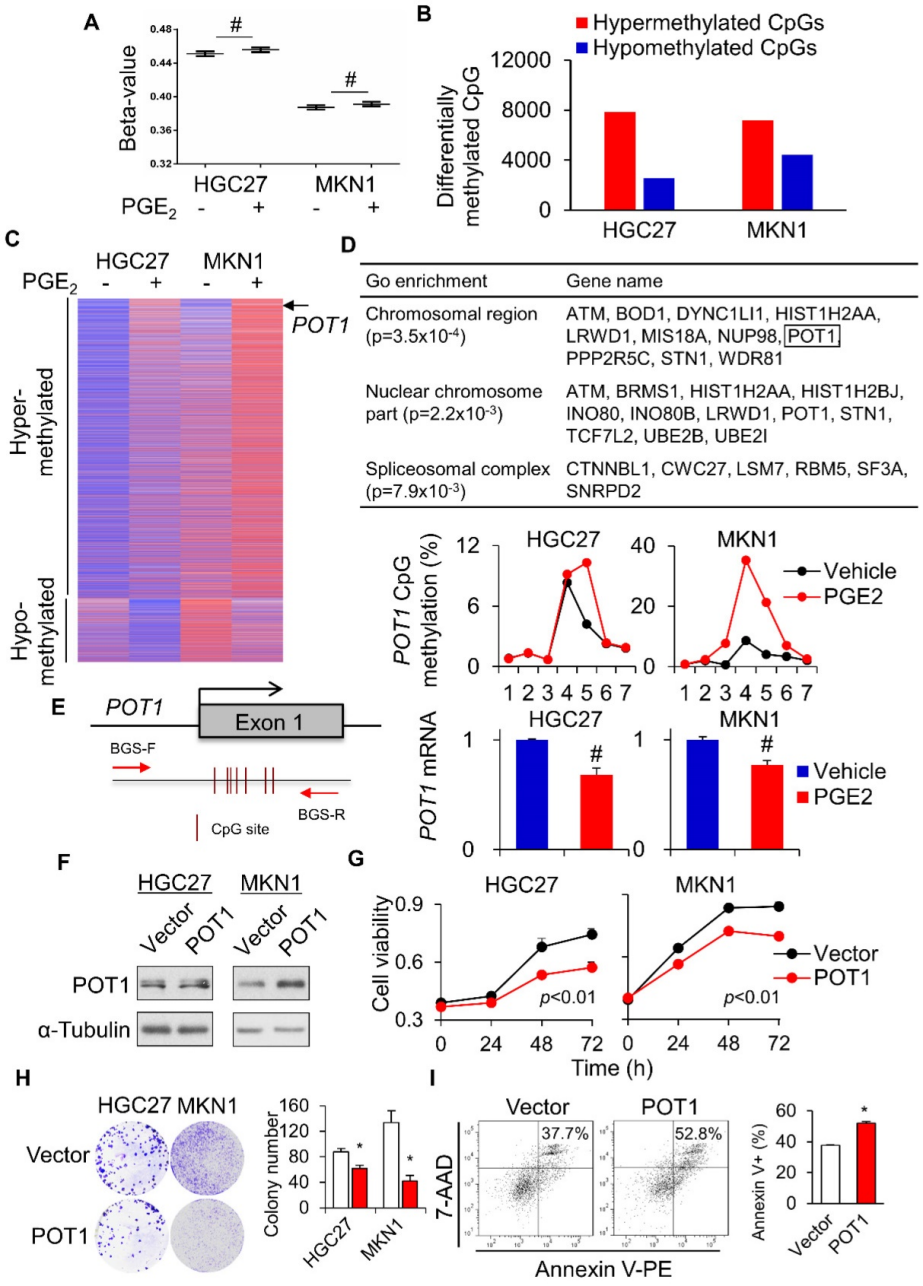
Global profiling of PGE_2_-induced DNA methylation. HGC27 and MKN1 cells treated with PGE_2_ for 48 h and their global DNA methylation profiles were assessed by Illumina 450K methylation array. (a) PGE_2_ treatment increased the overall methylation level (β-value) in all cell lines. (b) PGE_2_ induced hypermethylated loci in GC cells is greater than that of hypomethylated loci. (c) Commonly hypermethylated and hypromethylated genes in HGC27 and MKN1 cells after PGE_2_ treatment. (d) GO enrichment (WebGestalt) identified 3 biological process, chromosomal region, nuclear chromosome and spliceosomal complex, that were associated with hypermethylated loci. (e) *POT1* promoter was methylated by PGE_2_, leading to silencing of POT1 mRNA expression (f) *POT1* was overexpressed in HGC27 and MKN1 cells. (g and h) *POT1* overexpression inhibited GC cell proliferation, as indicated by MTT and colony formation assays. (i) POT1 overexpression induced apoptosis in MKN1 cells.

To further evaluate the impact of PGE_2_-induced DNA methylation on the global scale, we used the Illumina HumanMethylation 450K Array to survey the methylation status of over 485,000 CpG sites in 2 gastric cell lines (HGC27 and MKN1) treated with vehicle or PGE_2_ (2µM) for 48h. In all cell lines, PGE_2_ triggered an incremental rise in the overall methylation (beta-value) across 485K CpG sites (**Figure 5A**). Using a threshold of 1.5-fold change, the number of hypermethylated CpGs after treatment with PGE_2_ was 3.1- and 1.6-fold higher than hypomethylated CpGs in HGC27 and MKN1 cells, respectively (**Figure 5B**). Genes commonly hyper- or hypo-methylated in both cell lines were shown in **Figure 5C**. We also analyzed the β-values with DiMmer package 19. Hypermethylated genes identified by DiMmer were analyzed by GO enrichment analysis, revealing 3 biological processes involved (**Figure 5D**) that are associated with chromosomal or spliceosomal regulation. We next validated one candidate gene, *POT1*, which was hypermethylated by PGE_2_. BGS analysis confirmed increased *POT1* promoter methylation upon PGE_2_ treatment in both cell lines, with a corresponding decrease in *POT1* mRNA levels (**Figure 5E**). To evaluate the functional significance of COX-2-mediated silencing of *POT1*, we performed cell viability assays using GC cell lines. POT1 overexpression (**Figure 5F**) in HGC27 and MKN1 cells significantly suppressed cell viability as determined by MTT (**Figure 5G**) and colony formation assays (**Figure 5H**). Moreover, overexpression of POT1 significantly induced apoptosis in MKN1 cells (**Figure 5I**). These data suggest that COX-2/PGE_2_ may induce promoter hypermethylation of tumor suppressive genes in GC, thereby promoting gastric tumorigenesis.

### Inhibition of COX-2 and DNMT synergistically inhibit GC cell growth

**Figure 6:**
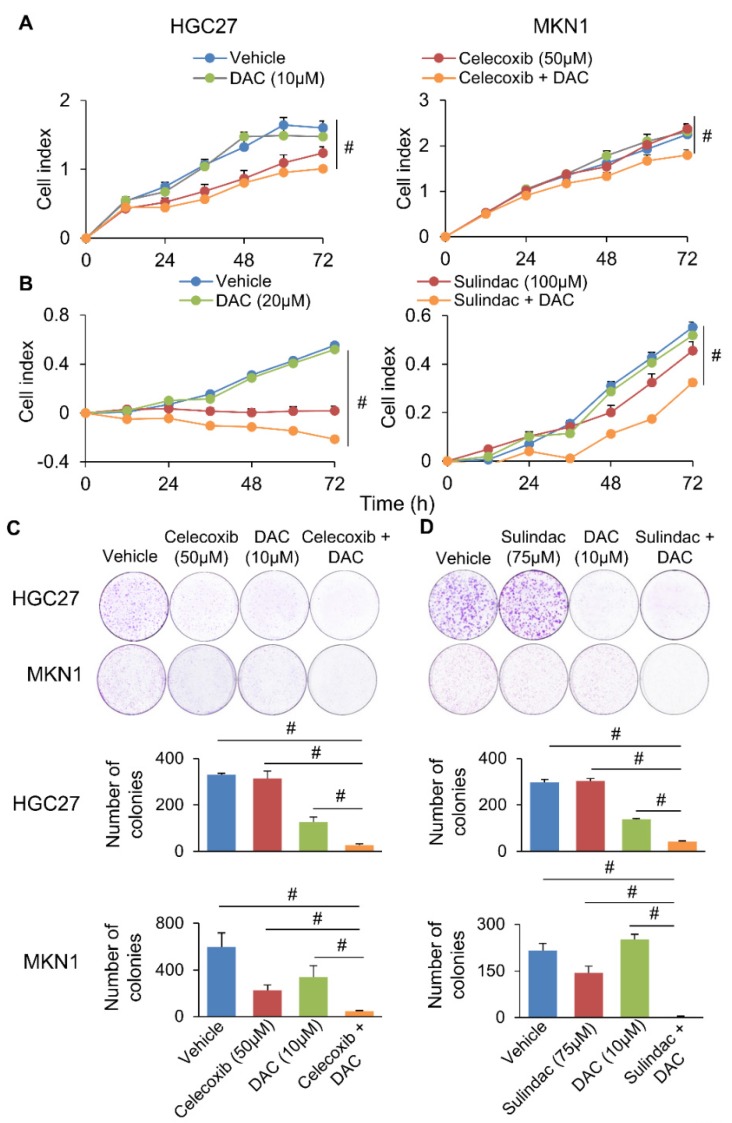
Simultaneous blockade of COX-2 (Celecoxib/Sulindac) and DNMT (Decitabine) synergistically inhibited gastric cancer growth. (a and b) Effect of Celecoxib/Sulindac, Decitabine or their combination on HGC27 and MKN1 cell proliferation, as assessed by cell growth curve assay. (c and d) Celecoxib/Sulindac, Decitabine or their combination on HGC27 and MKN1 cell apoptosis. In both assays, the drug combination more effectively inhibited cell growth as compared to single agents (*p*<0.0001).

**Figure 7:**
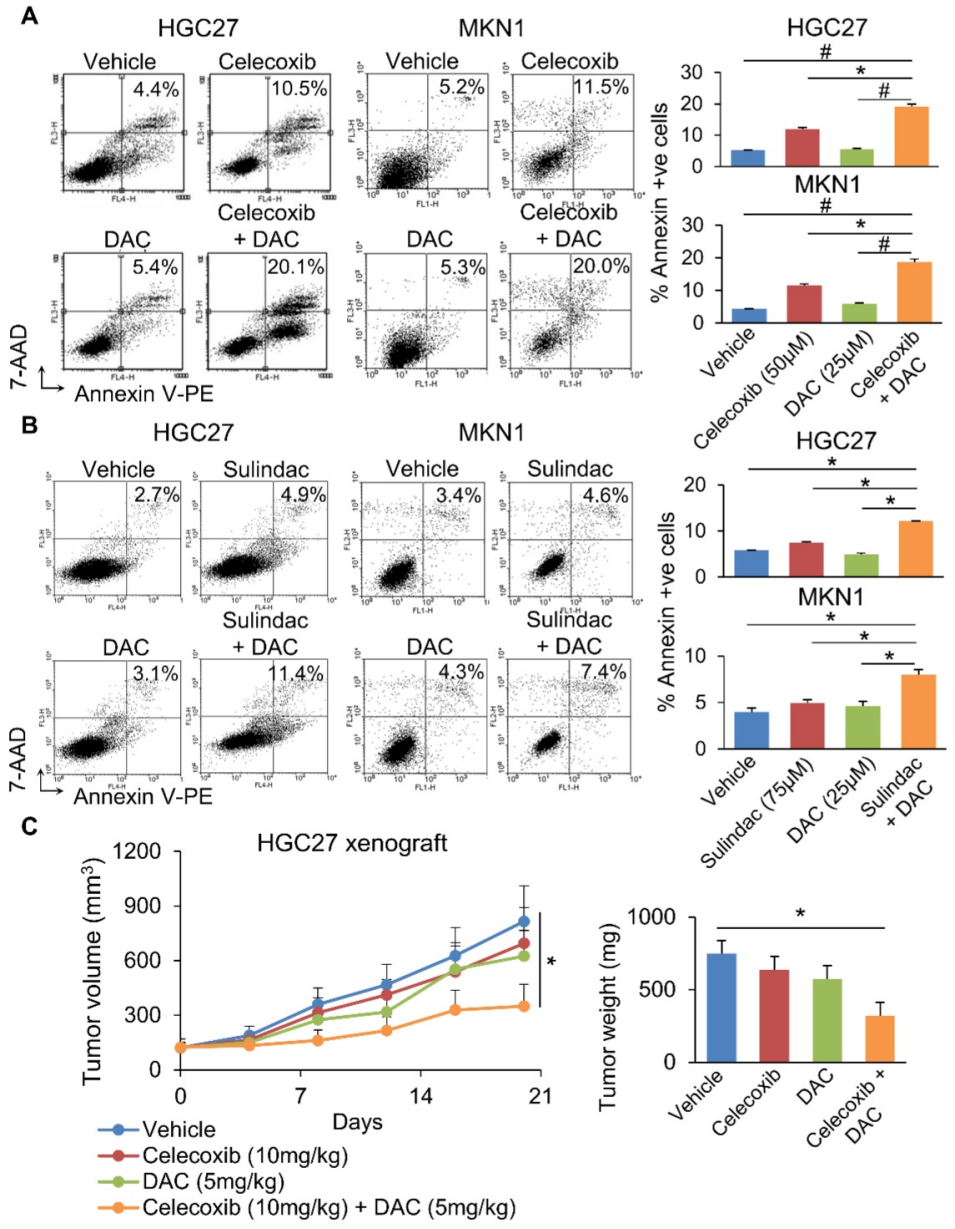
(a) Celecoxib or (b) Sulindac plus Decitabine induced apoptosis in HGC27 and MKN1 cells more potently compared to either drug alone. (c) Combination of Celecoxib (10mg/kg) and Decitabine (5mg/kg) synergistically inhibited the growth HGC27 xenografts in nude mice (*p*<0.03).

We next questioned whether co-inhibition of COX-2 and DNMT can synergistically impair GC growth. To evaluate this possibility, we combined Decitabine (5-aza-2'-deoxycytidine, a DNMT inhibitor) with Celecoxib (COX-2 specific inhibitor) or Sulindac (nonselective COX inhibitor) in HGC27 and MKN1 cells. Celecoxib and Sulindac inhibited growth of HGC27 cells with 72h-IC_50_ of 129 and 38μM; and MKN1 cells with 72h-IC_50_ of 88 and 69μM, respectively. Co-incubation with a non-cytotoxic dose of Decitabine (20μM) synergized with either COX inhibitor to reduce cell viability in HGC27 and MKN1 cells, as indicated by MTT assay (**Supplementary Figure 5**). Cell growth curve (xCELLigence) assay also revealed that Decitabine synergized with Celecoxib (**Figure 6A**) or Sulindac (**Figure 6B**) to inhibit cell growth in both GC cell lines. Colony formation assay showed that combining Decitabine with Celecoxib (**Figure 6C**) or Sulindac (**Figure 6D**) synergistically suppressed colony formation ability by over 80% and was superior to either agents given alone. These data indicate that co-inhibition of COX-2 and DNMT can synergistically suppress GC growth. To further evaluate the mechanism underlying their synergism, we determined apoptosis in HGC27 and MKN1 cells. As shown in **Figure 7A and 7B**, Celecoxib treatment alone (50μM) induced apoptosis by around 2-fold. The combination of Celecoxib with Decitabine induced apoptosis by 4-fold, representing a 2-fold enhancement in their potency in both cell lines (**Figure 7A and 7B**). In both cell lines, either Sulindac (75μM) or Decitabine (20μM) alone did not induced apoptosis. However, combination treatment increased the number of annexin V^+^ cells by ~2-fold (**Figure 7B**). We did not observe synergy between Sulindac/Celecoxib and Decitabine with respect to cell cycle, suggesting that this drug combination inhibits cell growth through increased induction of apoptosis.

### Celecoxib synergized with Decitabine to inhibit the growth of HGC27 xenografts

Given the synergistic effect of Celecoxib with Decitabine, we next questioned whether this approach is effective in suppressing tumorigenesis *in vivo*. HGC27 xenografts were implanted into nude mice. When tumor size reached ~120mm^3^, mice were randomized and treated with Celecoxib (10mg/kg), Decitabine (5mg/kg) or a combination of both, for 21 days. At the end point, neither Celecoxib nor Decitabine alone significantly inhibited HGC27 xenograft growth. The combination treatment, on the other hand, inhibited tumor growth by 68 % (*p*<0.05) (**Figure 7C**), indicating that Celecoxib and decitabine synergize to inhibit tumor growth *in vivo*.

## Discussion

Aberrant promoter DNA hypermethylation has been widely implicated in various stages of GC including cancer initiation, progression and cancer metastasis 20. The mechanism by which DNA methylation patterns are perturbed, however, are not well understood. In this study, we established a novel role of PGE_2_ in the induction of DNA methylation in GC. PGE_2_ administration induced the expression and activity of DNMT3B in a panel of GC cells, which in turn mediates the aberrant DNA promoter hypermethylation of tumor suppressor genes. *In vivo*, COX-2 transgenic expression also significantly induced the expression and activity of DNMT3B, which in turn, promoted global DNA methylation and promoter methylation of tumor suppressor genes. Moreover, pharmacological blockade of COX-2 in humans suppressed DNA methylation. Finally, the combinatorial targeting of COX-2 and DNMTs exerted a synergistic effect on GC cell growth, suggesting that such a regime can be used for the prevention or treatment of GC.

COX-2-derived PGE_2_ have been widely implicated in human carcinogenesis via various mechanisms, including induction of sustained inflammation in tumor microenvironment 21 and the activation of oncogenic signaling cascades such as RAS-MEK-ERK 22 and Wnt-β-catenin signaling 23. Overexpression of the COX-2/PGE_2_ axis can be detected in *H. pylori*-associated chronic gastritis, intestinal metaplasia and in GC 24. Here, we demonstrated that COX-2/PGE_2_ contributes to gastric carcinogenesis, at least in part, by inducing DNMT3B expression, as evidenced by 1) PGE_2_ treatment directly enhances DNMT3B expression and activity in gastric normal and cancer cells *in vitro*; 2) transgenic COX-2 expressing mice have elevated expression of DNMT3B and activity *in vivo*; and 3) the significant correlation between COX-2 and DNMT3B expression in human GC. Up-regulation of DNMT3B have key implications for aberrant DNA methylation. DNMT3B possessed *de novo* DNA methylation activity, is expressed in undifferentiated embryonic stem (ES) cells and is critical for mammalian development while being largely silenced in adult tissues 25. DNMT3B is overexpressed in GC and it cooperates with DNMT1 to silence tumor suppressor genes in cancer 26. Similar to our findings, DNMT3B is selectively up-regulated in Epstein-Barr virus (EBV)-associated GC 27, a subtype of GC characterized by extremely high levels of DNA hypermethylation, suggesting that induction of DNMT3B by PGE_2_ may be associated with an increased DNA methylation.

In GC, a dense methylation of promoter DNA is frequently observed in tumor suppressor genes related to DNA repair. COX-2/PGE_2_ induces DNA hypermethylation *in vitro* and *in vivo*, as indicated by the increased 5mC levels upon incubation of PGE_2_ in GC cells and COX-2 transgenic expression in mice, respectively. Our intestinal metaplasia patient cohort also showed that COX-2 inhibitor (rofecoxib) treatment slowed DNA hypermethylation in these patients. Hypermethylation of tumor suppressor gene promoters is a key hallmark of GC. Two such classical tumor suppressor silenced via promoter hypermethylation are *MGMT* and *CNR1*. We observed hypermethylation of *MGMT* and *CNR1* promoters upon PGE_2_ treatment in GC cell lines. Whilst Xia *et al*. 16 also demonstrated MGMT and CNR1 hypermethylation by PGE_2_ in colorectal cancer cells, our study was the first to use COX-2 transgenic mice to confirm *MGMT* promoter methylation in gastric tissues by COX-2/PGE_2_ axis *in vivo*. Genome-wide methylation profiling revealed chromosome-associated candidate genes that are hypermethylated by PGE_2_. Validation of top candidate gene, *POT1,* revealed that it is hypermethylated by PGE_2_ in GC cells. Ectopic *POT1* expression in GC cells suppressed cell viability and induced apoptosis, suggesting that it functions as a tumor suppressor in GC. We and others have shown that the DNA methylation of tumor suppressors, such as SCNN1B, CAB39L and PKNOX2, are associated with gastric carcinogenesis and predict poor prognosis in GC patients 8, 9, 28. These data further indicate that COX-2-derived PGE_2_ may play an active role in gastric tumorigenesis through DNA methylation.

Drug combination is a promising approach to synergistically enhance chemotherapeutic effect(s) whilst minimizing potential toxicity. Given that COX-2/PGE_2_ axis is important for aberrant DNA methylation, it provides a rationale to combine the inhibitors of COX-2 and DNMTs to simultaneous target the expression and activity of DNMTs. We evaluated the use of Sulindac (non-selective COX inhibitor) and Celecoxib (COX-2 specific inhibitor) in combination with Decitabine (DNMT inhibitor) in GC cell lines. It was shown that either COX-2 specific inhibitor Celecoxib or non-selective COX-2 inhibitor Sulindac synergizes with decitabine to inhibit GC in* in vitro* and *in vivo*, suggesting that targeting PGE_2_-DNMT axis represents a novel approach that may be used in prevention and treatment of GC.

In summary, our findings uncovered the role of COX-2/PGE_2_ axis as an inducer of DNA hypermethylation in GC *in vitro* and *in vivo*, which leads to silencing of tumor suppressor genes thereby promoting gastric carcinogenesis. Targeting of COX-2 could reduce DNA methylation and it synergizes with DNMT inhibition to suppress GC.

## Materials and Methods

### Cell culture

Nine human gastric cancer cell lines (AGS, BGC823, HGC27, KATOIII, MKN1, MKN28, NCI-N87, SNU-1 and TMK-1) and one normal GES-1 gastric cell line were used in this study. Cell lines were maintained in RPMI-1640 medium (Gibco, Rockville, MD) with 10% fetal bovine serum (Life Technologies, Carlsbad, CA), penicillin (50 unit/ml) and streptomycin (50 µg/ml) at 37^0^C with 5% CO_2_.

### Human gastric tissues

Primary tumor and their adjacent non-tumor tissues were obtained from gastric cancer patients during operation prior to any therapeutic intervention from the Prince of Wales hospital, Hong Kong. All samples were subsequently verified by histological evaluation. Informed consent was given to all the patients. The study protocol was approved by the Clinical Research Ethics Committee of Chinese University of Hong Kong.

### RNA extraction and real-time PCR analyses

Total RNA was extracted from cells and tissues using the Direct-zol^TM^ RNA MiniPrep kit (Zymo Research, Irvine, CA). cDNA was synthesized from RNA using the High-Capacity cDNA Reverse Transcription Kit (Life Technologies). Real-time PCR was performed in a Roche LightCycler 480 system. Primer sequences are listed in **Supplementary Table 1**.

### Bisulfite genomic sequencing (BGS)

Genomic DNA was extracted using the QIAamp DNA Mini kit (Qiagen, Hilden, Germany). DNA (500ng) was bisulfite modified with the EZ DNA Methylation^TM^ Kit (Zymo Research). The bisulfite-modified DNA was amplified using primers for bisulfite genomic sequencing (BGS). The primers were designed using MethPrimer (http://www.urogene.org/methprim er/index1.html) **Supplementary Table 1**. PCR products for BGS were confirmed by agarose gel electrophoresis and then directly sequenced. Sequence data were analyzed by Chromas software.

### Cell viability analysis

Cell viability was determined using the MTT assay (Sigma-Aldrich, St Louis, MO). 5x10^3^ cells/well were seeded in a 96-well plate and incubated overnight. At the end of drug treatment, MTT (0.5mg/ml) was added and incubated at 37^0^C for 4h. The reaction was stopped by 10% SDS in 0.01N HCl and absorbance at 570nm was measured.

### Colony formation assay

Cells (1x10^3^ cells/well) were seeded in a 6-well plate and incubated overnight. Then the drugs at the indicated concentrations were added and incubated for 1 week. Colonies were fixed with 70% ethanol, stained with crystal violet solution, and counted.

### Cytokinetic analysis

Apoptosis was determined by staining cells with Annexin V and 7-amino-actinomycin (7-AAD) (BD Biosciences) with flow cytometry analysis. Cell cycle analysis was performed on fixed cells stained with propidium iodide.

### Illumina 450K methylation array analysis

HGC27 and MKN1 cells (1x10^6^ cells) were treated with vehicle or PGE_2_ (2x10^-6^ M) for 48h. DNA was extracted using the QIAamp DNA Mini kit. We then submitted the samples to Shanghai Biotechnology Corporation (Shanghai, China) for Illumina 450K methylation array analysis. Background subtraction and normalization were performed. DiMmer package was used to identify commonly methylated genes in gastric cell lines. Genes epigenetically altered were included in Gene Ontology (GO) enrichment analysis using Gene Set Analysis Toolkit V2 (http://bioinfo.vanderbilt.edu/webgestalt/), using all genes from humans as reference. Hypergeometric test statistical method and the BH multiple test adjustment method were used. Pathways with at >4 genes and adjusted *p*-values <0.05 were considered significantly enriched.

### SiRNA-mediated knockdown

Control siRNA and siRNA targeting the Prostaglandin E Receptors (EP) 1, 2 and 4 were purchased from RiboBio, Inc. (Guangzhou, China). SiRNAs were transfected into gastric cancer cells using Lipofectamine 2000 according to the manufacturer's protocol.

### COX-2 transgenic mice

COX-2 transgenic mice were generated as described 17. Wild-type (WT) littermates were analyzed in parallel as controls. All C57BL/6 mice were maintained in metal cages on a 12 h light/dark cycle. The study protocol was approved by the Animal Experimentation Ethics Committee of the Chinese University of Hong Kong and all the experiments were performed in accordance with local guidelines. GC was induced in COX-2 transgenic and WT mice using N-methyl-N-nitrosourea (MNU) as described. All the animals were killed at the end of week 50 by cervical dislocation. The stomach was opened along the greater curvature, examined macroscopically and fixed in 10% buffered formalin or snap frozen for subsequent histological and molecular analysis.

### Xenograft study

This animal study was approved by the Animal Experimentation Ethics Committee of the Chinese University of Hong Kong. HGC27 cells (1x10^7^) were injected into the right flank of female nude mice (7-8 weeks old). When tumor volume reached 500mm^3^, the tumor was cut into 1mm^3^ pieces and implanted into both flanks of 6-7 weeks female nude mice (*n*=20). When average tumor size reached 150mm^3^, the mice (*n*=5 per group) were randomized into four groups and given 1) vehicle, 2) Celecoxib (10mg/kg/d, oral gavage), 3) decitabine (1mg/kg, i.p., 3 per week), and 4) Celecoxib (10mg/kg/d, oral gavage) plus decitabine (1mg/kg, i.p., 3 per week). Tumor volumes were measured twice per week with a digital caliper and tumor volumes were calculated by as follow: tumor volume=[length×width× (length+width/2)×0.5]. After 3 weeks of treatment, the animals were sacrificed, and the tumors were collected and stored at -80^0^C.

### 5-mC staining by immunohistochemistry

A randomized, placebo-controlled trial was conducted (from 2001 to 2004) consisting of patients initially diagnosed with gastric intestinal metaplasia 18. Patients were randomized and treated with placebo or rofecoxib 25 mg daily for 2 years. Age- and gender-matched samples from the placebo and rofecoxib groups (N=42) were randomly selected. Paraffin blocks were retrieved, section and stained with 5mC antibody (clone clone 33D3). Slides were scored by a senior pathologist blinded to the nature of the samples tested.

### Western blot analysis

Total and/or nuclear protein was separated by sodium dodecyl sulfate-polyacrylamide gel electrophoresis and transferred onto nitrocellulose membranes. Blots were probed with primary antibodies at 4^0^C overnight, and then with secondary antibody at room temperature for 1h. Proteins of interest were visualized using ECL Plus Western blotting detection reagents (GE Healthcare, Piscataway, NJ).

### DNMT3B activity assay

DNMT3B activity in cells was evaluated using the EpiSeeker DNMT3B assay kit (Abcam, Cambridge, MA). The assay was performed according to the manufacturer's instructions and 10 micrograms of nuclear protein was used in each test.

### Dot Blot

Genomic DNA was isolated using commercially available kit, and diluted DNA were spotted on nitrocellulose membrane. After drying, nitrocellulose membrane was crosslinked under UV light and probed with anti-5mC antibody. Anti-ss/dsDNA served as loading control.

### Statistical analysis

Data are presented as means±S.D. Difference between two groups (normally distributed) was determined by the Student's t-test. Non-parametric data between two groups were computed by Chi-square test or Fisher Exact test. *P*<0.05 were considered statistically significant.

## Supplementary Material

Supplementary figures.

## Abbreviations

5mC: 5-Methylcytosine
BGS: Bisulfite genomic sequencing
CIMP: CpG island methylator phenotype
COX-2: Cyclooxygenase-2
EP: E-prostanoid receptors
GC: Gastric cancer
MNU: N-methyl-N-nitrosourea
NSAIDs: Nonsteroidal anti-inflammatory drugs
PGE_2_: Prostaglandin E_2_
