# Supplementary material for: Prostaglandin E2 induces DNA hypermethylation in gastric cancer in vitro and in vivo
Source: Theranostics. 2019 Aug 14;9(21):6256–68. doi: 10.7150/thno.35766 (PMC6735505; doi:10.7150/thno.35766)
Supplement: Supplementary file 1 — Supplementary figures. [file thnov09p6256s1.pdf]

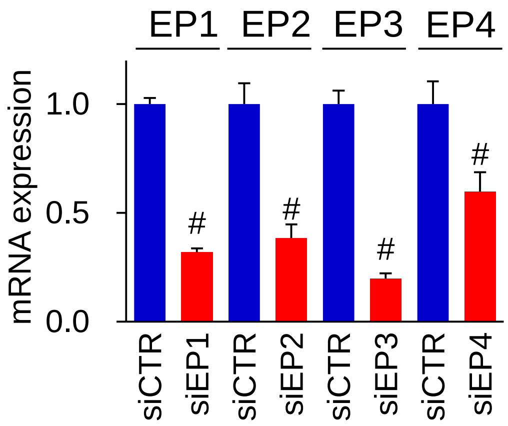

Supplementary Figure 1. qPCR validation of siRNA-mediated knockdown of EP receptors in HGC27 cells.

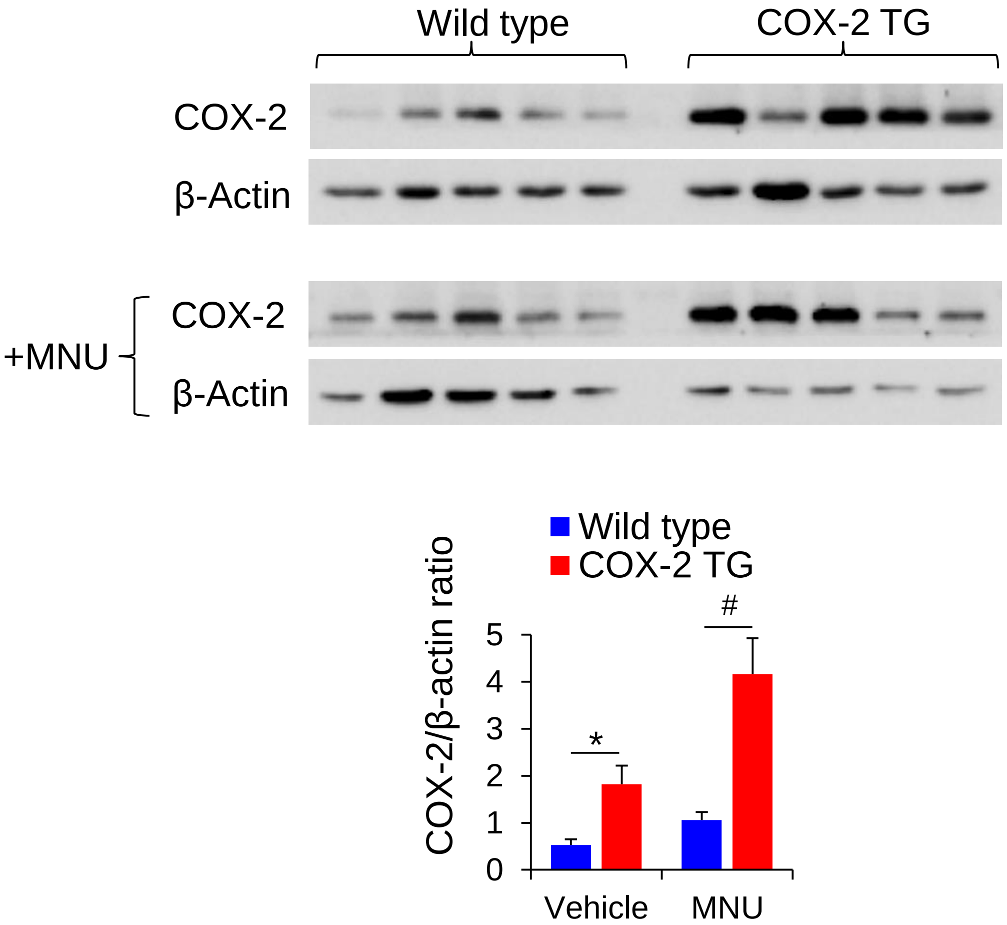

Supplementary Figure 2. Western blot confirmed overexpression of COX-2 protein in COX-2 TG mice.

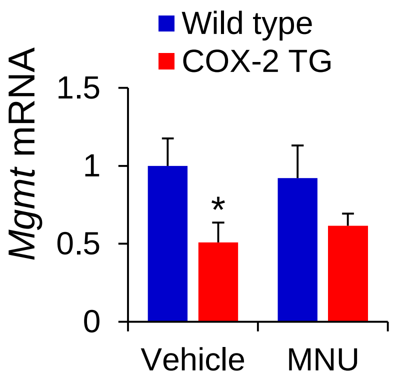

Supplementary Figure 3. MGMT mRNA was silenced in COX-2 TG mice.

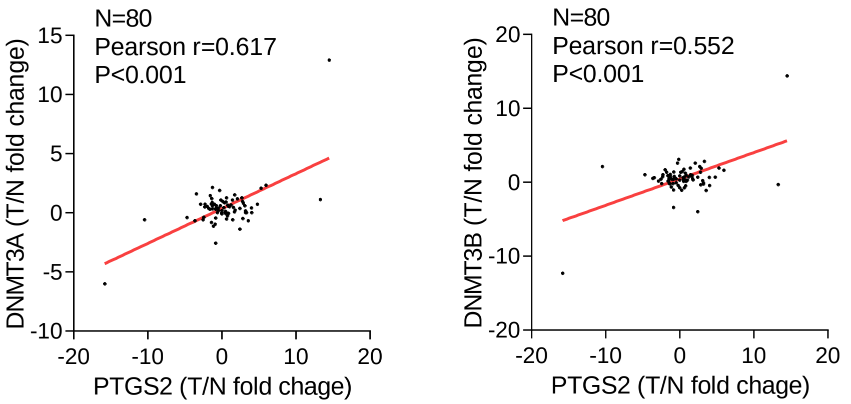

Supplementary Figure 4. DNMT3A and DNMT3B mRNA were positively correlated with PTGS2 mRNA in human GC (GSE27342).

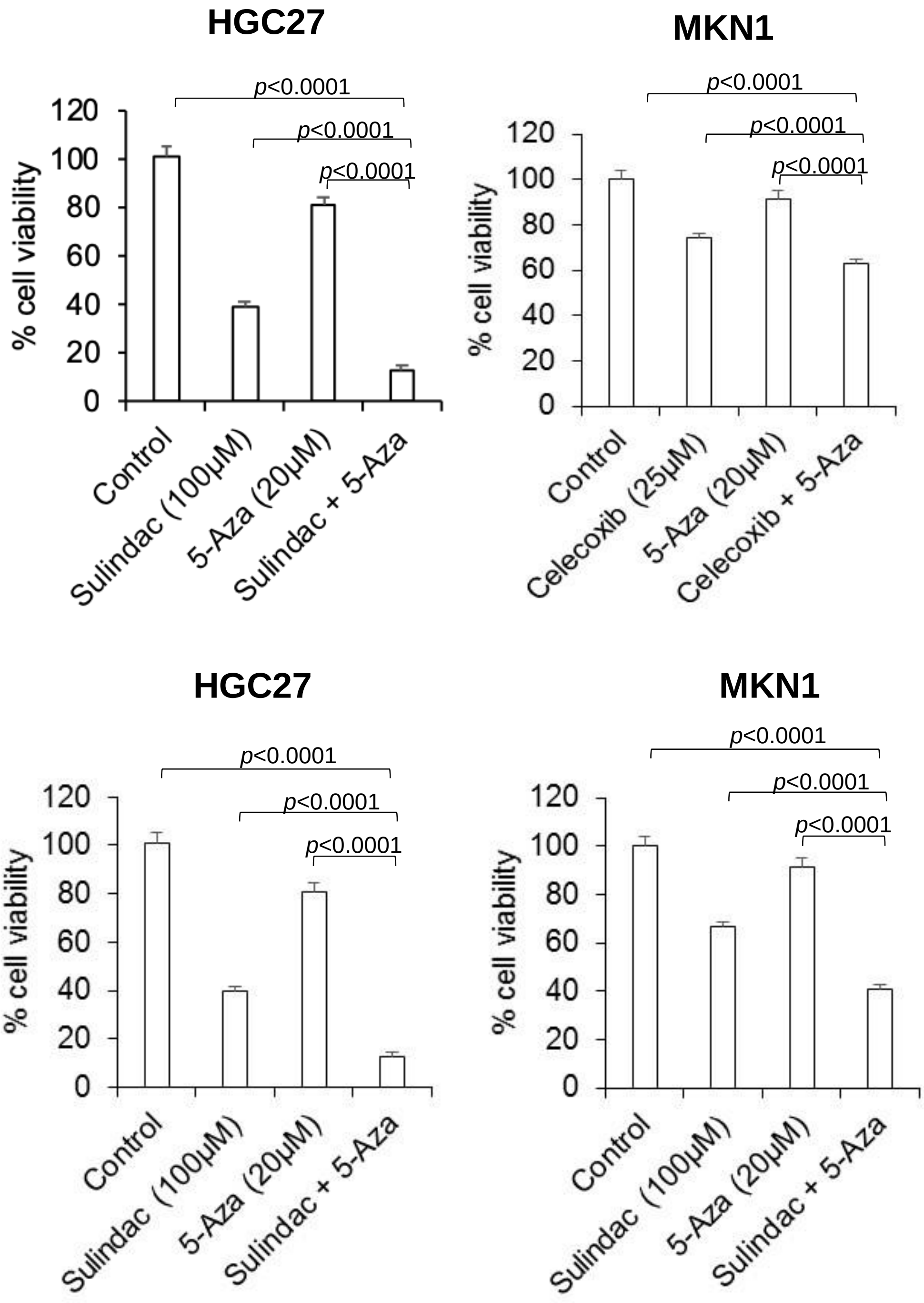

Supplementary Figure 5. Synergy between sulindac/celecoxib and 5-Aza in GC cell lines.
